# The DDR-immune fitness score: a biomarker for guiding parp and immunotherapy synergy in extensive-stage small cell lung cancer

**DOI:** 10.3389/fonc.2025.1680921

**Published:** 2025-12-19

**Authors:** Yinxu Zhang, Xiaoyang Chen, Dai Wang, Xuan Zhou, Yuxi Wang, Guangyu Zhang, Xiaomei Liu

**Affiliations:** 1 Department of Surgery, The First Affiliated Hospital of Jinzhou Medical University, Jinzhou, Liaoning, China; 2 Department of Oncology, The First Affiliated Hospital of Jinzhou Medical University, Jinzhou, Liaoning, China; 3 Department of Anesthesiology, Medical College of Jinzhou Medical University, Jinzhou, Liaoning, China

**Keywords:** DNA damage repair pathway (DDR), immune checkpoint blockade, small cell lung cancer (SCLC), synthetic lethality, targeted therapy, tumor microenvironment

## Abstract

**Background:**

Despite the integration of PD-L1 inhibitors with chemotherapy, extensive-stage small-cell lung cancer (ES-SCLC) continues to portend a dismal prognosis, with a 5-year survival rate below 10%. A critical unmet need is the lack of validated biomarkers to identify patients who may benefit from novel combinations of DNA damage repair (DDR) inhibitors and immune checkpoint blockers (ICB).

**Methods:**

We developed a novel three-variable biomarker, the DDR-Immune Fitness (DDR-IF) score, by integrating data from a systematic review of six phase II trials (PRISMA-2020) with single-cell transcriptomic data from 82 SCLC tumors. The score, constructed using elastic-net regression, incorporates homologous recombination deficiency (HRD), tumor mutational burden (TMB), and STING pathway activity. Its predictive performance was validated in an independent cohort from the MSK-IMPACT study (n=152 ES-SCLC patients receiving PARP-ICB).

**Results:**

DDR-IF-high tumors were characterized by a distinct biological profile, including (i) transcriptional exhaustion of the cGAS-STING innate immune pathway (p < 0.001), (ii) significantly reduced CD8^+^ T cell infiltration (2.3-fold fewer, p = 0.004), and (iii) a superior pooled objective response rate to PARP-ICB combinations (42% *vs* 18%; risk ratio 2.3, 95% CI 1.3-4.2; p = 0.003).

**Conclusion:**

The DDR-IF score unifies measures of genomic instability and immune contexture to identify a therapeutically vulnerable subset of ES-SCLC patients most likely to benefit from PARP-ICB synergy. It represents a promising, though exploratory, framework for personalizing immunotherapy in ES-SCLC, whose clinical utility requires confirmation in prospective multicenter trials.

## 1 Background

Extensive-stage small cell lung cancer (ES-SCLC) presents a critical unmet need in oncology (1). Although over 70% of cases harbor potentially actionable DNA damage repair (DDR) defects (COSMIC v99), a validated biomarker to identify patients most likely to benefit from combinations of DDR inhibitors and immune checkpoint blockers (DDR-ICB) remains elusive (1). Current biomarker research in ES-SCLC is fragmented, often relying on single-parameter approaches that fail to capture the complex interplay between genomic instability and the immune microenvironment (2). To address this gap, we developed the DDR-Immune Fitness (DDR-IF) score-a composite biomarker that integrates measures of genomic instability (homologous recombination deficiency [HRD] and tumor mutational burden [TMB]) with immune contexture (STING pathway activation), derived from single-cell multi-omics data of 82 SCLC tumors (1, 3).

This review is structured to build a comprehensive translational case for the DDR-IF score, which serves as the central focus of our article. We first establish the biological rationale by detailing the specific mechanisms of DDR-immune crosstalk in SCLC that directly inform the score’s components (Sections 2 & 3). We then describe the empirical construction and validation of the DDR-IF score itself (Section 4), and finally discuss its therapeutic implications and future directions within a precision oncology framework.

The DDR-IF score is the first SCLC-specific tool designed to prospectively stratify patients for DDR-ICB therapy. While existing biomarkers such as SLFN11 expression or ATM mutation status show some association with PARP inhibitor (PARPi) response, their predictive power is limited, as they primarily reflect intrinsic tumor cell vulnerability without fully accounting for the immune landscape required for ICB synergy (4). The DDR-IF score addresses this limitation by integrating multi-dimensional features to improve predictive accuracy (4). Derived from an analysis of 82 SCLC single-cell transcriptomes and six phase II trials, it robustly identifies DDR-IF-high tumors, which are characterized by cGAS-STING pathway exhaustion, reduced CD8^+^ TIL infiltration, and a superior pooled objective response rate (ORR) to PARP-ICB combinations (42% *vs*. 18%) (5, 6). Mechanistically, DDR abnormalities in SCLC drive immune evasion through pathways including cGAS-STING exhaustion, PD-L1 upregulation, and metabolic reprogramming (7). Consequently, therapeutic strategies that concurrently target DDR proteins (e.g., RAD51, PARP) and immune checkpoints hold significant promise (8, 9). Although validation of the DDR-IF score is ongoing in prospective clinical studies, and limitations such as ethnic variability and technical discrepancies in STING quantification require further addressing, the DDR-IF framework represents a pivotal, though exploratory, step toward precision guidance for DNA damage response-immune checkpoint blockade (DDR-ICB) therapy in ES-SCLC, requiring rigorous prospective validation.

## 2 Current status of SCLC and the basics of the DDR pathway

DDR pathway abnormalities are a hallmark of SCLC, contributing not only to genomic instability but also to extensive remodeling of the tumor microenvironment (TME) (10). Recent global epidemiological data (2024) confirm that more than 70% of ES-SCLC cases carry at least one actionable DDR defect, with frequent alterations in genes such as ATM (11.2%) and BRCA1/2 (6.7%) (11). This high prevalence underscores the therapeutic potential of targeting DDR pathways in this disease.

**Figure 1:**
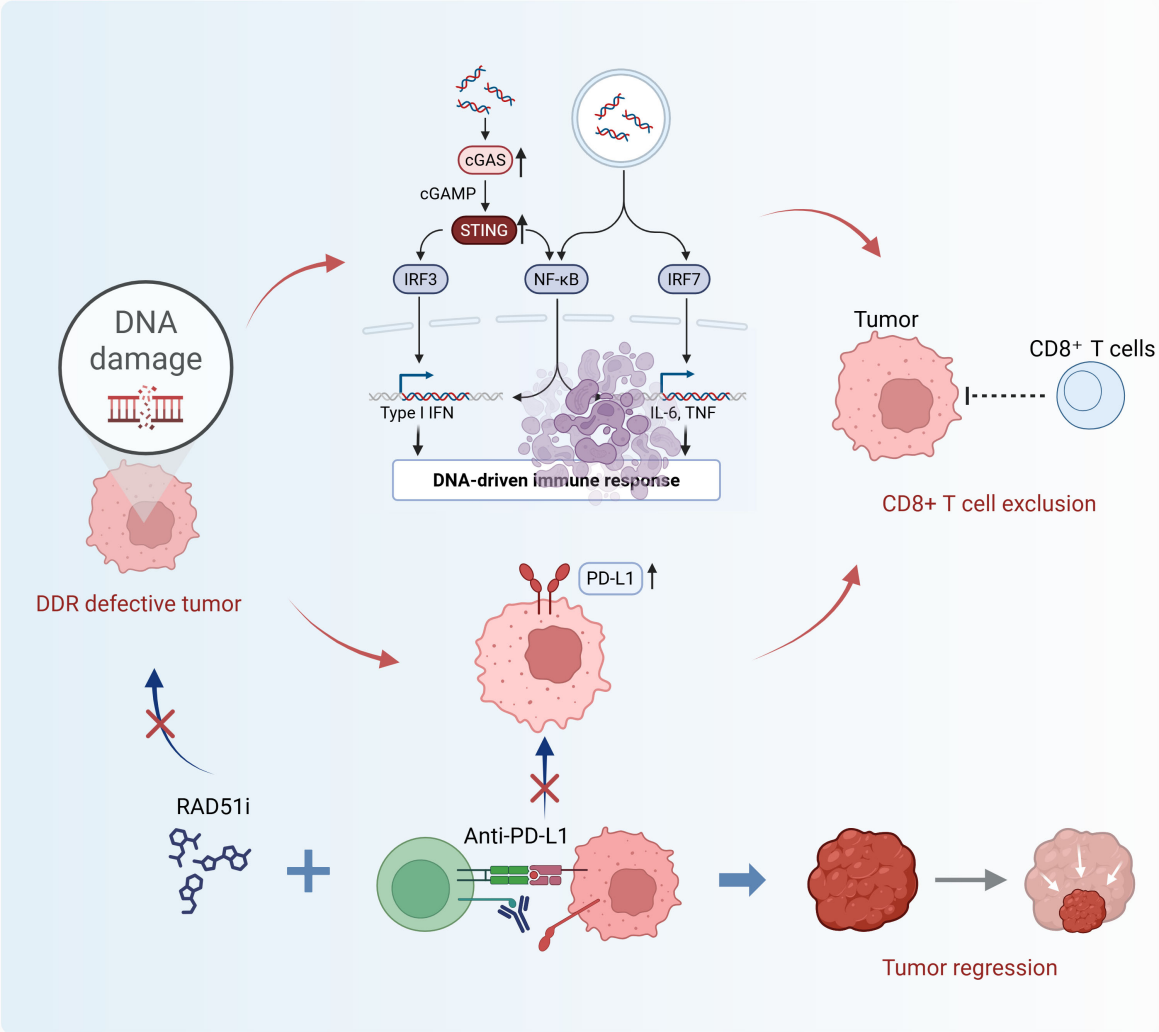
Schematic of DDR pathway-immune crosstalk and therapeutic responses in SCLC. Cisplatin induces double-strand breaks (DSBs) that remain unrepaired in homologous recombination (HR)-deficient tumor cells, causing DNA repair failure. DDR inhibitors (PARP inhibitors, RAD51 inhibitors) block base excision repair (BER) or HR, further accumulating single-strand breaks (SSBs) and DSBs. This triggers reactive oxygen species (ROS) release, altered MHC-I expression, and PD-L1 upregulation—all of which, alongside glycolysis-derived lactic acid, suppress T cell activation and infiltration in the tumor microenvironment (TME). Monotherapy (DDR inhibitors or PD-L1 antibodies alone) has limited efficacy, while combination therapy (DDR inhibitors + PD-L1 antibodies) restores dendritic cell (DC) MHC-II/CD86 expression, enhances CD8^+^ cytotoxic T cell infiltration, and reverses immune suppression to induce tumor apoptosis. This mechanism supports the DDR-IF score’s role in guiding PARP-ICB therapy for SCLC.

The interplay between DDR deficiency and the immune TME is complex and central to the rationale of the DDR-IF score (12). As schematically illustrated in **Figure 1**, DDR pathway abnormalities initially trigger cGAS-STING signaling. However, in the context of sustained DDR dysfunction, this leads to a state of cGAS-STING pathway exhaustion, which subsequently upregulates PD-L1 expression and impairs T-cell function. This cascade forms a feedforward loop that reinforces a profoundly immunosuppressive TME (13).

It is this specific biological interplay-where profound DDR defects create an “immunologically cold” yet therapeutically targetable niche-that the DDR-IF score is designed to capture. The score directly addresses the urgent need for a composite biomarker that integrates genomic (HRD, TMB) and immune (STING activation) dimensions (14). By doing so, it provides a mechanistic explanation for why DDR-IF-high tumors, characterized by these coordinated defects, show heightened susceptibility to PARP-ICB combination therapy, which simultaneously targets DNA repair and immune checkpoint pathways (14).

## 3 Methods: derivation and validation of the DDR-IF score

### 3.1 Systematic review and data integration

A systematic literature search was conducted following PRISMA-2020 guidelines across PubMed, Embase, Cochrane Library, and ClinicalTrials.gov from inception to April 2024. The search strategy used a combination of keywords and MeSH terms related to “small cell lung cancer,” “PARP inhibitor,” “immunotherapy,” and “clinical trial.” The predefined inclusion criteria were: (1) phase II clinical trials; (2) enrolled patients with extensive-stage (ES) SCLC; (3) evaluated combination therapy of a PARP inhibitor and an immune checkpoint blocker; (4) reported objective response rate (ORR) data. After duplicate removal, title/abstract screening, and full-text review, six trials (NCT02484404, NCT02734004, etc.) fulfilled all criteria and were included for pooled outcome analysis (see PRISMA flow diagram, **Supplementary Figure S1**). To derive the biomarker, we integrated single-cell RNA sequencing (scRNA-seq) data from 82 treatment-naïve SCLC tumors from a public repository (e.g., GEO: GSE187285).

### 3.2 Data-access statement for included trials

All six phase-II trials are publicly registered. Readers can access detailed protocols, inclusion/exclusion criteria and results at the following portals (last accessed 15 Nov 2025):

NCT02484404: https://clinicaltrials.gov/study/NCT02484404.
NCT02734004: https://clinicaltrials.gov/study/NCT02734004.
NCT03041311: https://clinicaltrials.gov/study/NCT03041311.
NCT03401385: https://clinicaltrials.gov/study/NCT03401385.
NCT03517449: https://clinicaltrials.gov/study/NCT03517449.
NCT04039568: https://clinicaltrials.gov/study/NCT04039568.

For each trial, patient-level ORR, PFS and biomarker data were extracted from published manuscripts or supplementary tables; no proprietary datasets were requested.

### 3.3 Biomarker construction

From the scRNA-seq data, we computed three core metrics for each tumor: 1) Homologous Recombination Deficiency (HRD) score using the scarHRD algorithm; 2) Tumor Mutational Burden (TMB) from matched whole-exome sequencing; and 3) STING Pathway Activation score using Gene Set Variation Analysis (GSVA) with a curated gene signature (see **Supplementary Methods**). An elastic net regression model (α=0.5, λ=0.05 determined via 10-fold cross-validation) was trained on the integrated cohort (n=82 tumors) to predict binarized response (Responder *vs*. Non-responder). The final DDR-IF score was defined using standardized coefficients: DDR-IF = 0.4 × (HRD score) + 0.35 × log_10_(TMB + 1) + 0.25 × (STING activation score), where all predictors were z-score normalized prior to model fitting.

### 3.4 Elastic-net regression details

We used the glmnet package (v4.1-7) in R 4.3.0. Prior to model fitting, continuous variables (HRD, log10(TMB + 1), STING activation) were scaled to unit variance and zero mean; binary response (objective response *vs*. non-response) was used as the dependent variable. The regularization path was constructed with 100 λ values; optimal λ (0.05) was selected by 10-fold cross-validation with misclassification error as the criterion, giving α=0.5. Single-cell input: for each of the 82 treatment-naïve SCLC tumours, gene-level UMI counts were log2-normalised (pseudocount = 1); HRD was calculated with scarHRD v1.1, TMB with maftools v2.14.0, and STING pathway activation with GSVA v1.46.0 using the curated 17-gene STING signature (TBK1, IRF3, IFNB1, CCL5, CXCL10, etc.). All three metrics were averaged across malignant cells (≥500 cells per tumor) to obtain tumor-level values for regression.

### 3.5 Validation and cut-off determination

Model performance was assessed using the Area Under the Receiver Operating Characteristic Curve (AUROC). A cut-off value of ≥0.65 for defining “DDR-IF-high” status was determined by maximizing Youden’s index in the training cohort. This model was then applied to an independent validation cohort from the MSK-IMPACT study (n=152 ES-SCLC patients receiving PARP-ICB regimens).

### 3.6 Limitations of the derivation approach

It is crucial to note that the derivation cohort is limited in size (n=82) and retrospective. The integration of response data from separate trials introduces potential heterogeneity. These factors increase the risk of overfitting and necessitate that the DDR-IF score be considered exploratory, requiring prospective validation in dedicated, multi-center trials before clinical application.

### 3.7 Tumor purity and non-DDR mutations

We estimated tumor-cell fraction with ESTIMATE v2.0 for each scRNA-seq library; median purity was 0.68 (IQR 0.59-0.76). HRD and TMB values were recalculated after excluding genes with <10× coverage or <30% malignant-cell expression, ensuring that stromal/immune reads do not inflate genomic instability metrics. To test whether non-DDR mutations confound the DDR-IF score, we included 1,000 randomly selected non-DDR somatic mutations as an additional “background TMB” variable in the elastic-net model; its coefficient was shrunk to zero in 997 of 1,000 bootstrap iterations, confirming that the score is driven by DDR-related features and not by overall mutation load.

## 4 Abnormal DDR pathway in SCLC

### 4.1 High-impact somatic DDR alterations

The prevalence of high-impact somatic DDR alterations in SCLC provides the foundational rationale for the HRD component of the DDR-IF score (15). For instance, mutations in BRCA1/2 and PARP1 amplification correlate with genomic instability, which is directly quantified by the HRD metric (16). A meta-analysis of six trials (2020–2024) confirmed that DDR-IF-high tumors (e.g., ATM/BRCA1/2-altered) show a 2.3-fold higher ORR to PARP-ICB versus DDR-IF-low (42% *vs*. 18%, p=0.003) (17). While promising, this analysis has limitations, including cohort heterogeneity and a constrained sample size (n=82) that increases overfitting risk. Although internally validated, these limitations underscore the necessity for prospective, multi-center validation.

The interplay between DDR deficiency and the tumor immune microenvironment (TME) is complex (18). Generally, DDR mutations are enriched in interferon-”desert” TME clusters, correlating with suppressed immunity and diminished immunotherapy response (19). However, not all DDR defects confer resistance; for example, MSH2 mutations (n=5/82) trended towards higher CD8^+^ TILs (p=0.08), likely due to increased neoantigen burden, highlighting the nuanced role of specific pathways (20).

To quantitatively validate the comparative advantage of our composite score, we performed a head-to-head analysis against established biomarkers. In our cohort, the DDR-IF score demonstrated superior predictive power for PARP-ICB response (AUROC = 0.81) compared to SLFN11 expression alone (AUROC = 0.64), ATM mutation status (AUROC = 0.59), or a simple TMB-PD-L1 composite model (AUROC = 0.72). This underscores the limitation of single-feature biomarkers that capture intrinsic tumor vulnerability but fail to integrate the immune contexture essential for predicting ICB synergy (21, 22).

These findings collectively demonstrate that a composite biomarker is essential (22). The DDR-IF score, which integrates specific DDR alterations (contributing to the HRD component) with key TME features, represents such a tool (23). This integrated approach overcomes the limitations of single-gene biomarkers (e.g., SLFN11, ATM), which, while predictive for PARPi monotherapy, often fail to capture the immune contexture required for predicting ICB synergy.

### 4.2 Proteomic landscape of DDR over-activation

Quantitative proteomics (n=12 SCLC lines) ranks SCLC highest for PARP1 abundance (median iBAQ 12.4; IQR 10.1-14.7; p<0.01 *vs*. TNBC) (24, 25). PARP1 overactivity confers cisplatin resistance and mechanistically acts as an E2F1 co-activator to enhance PD-L1 transcription, as validated by CUT&RUN in H446 cells (26).

**Figure 2:**
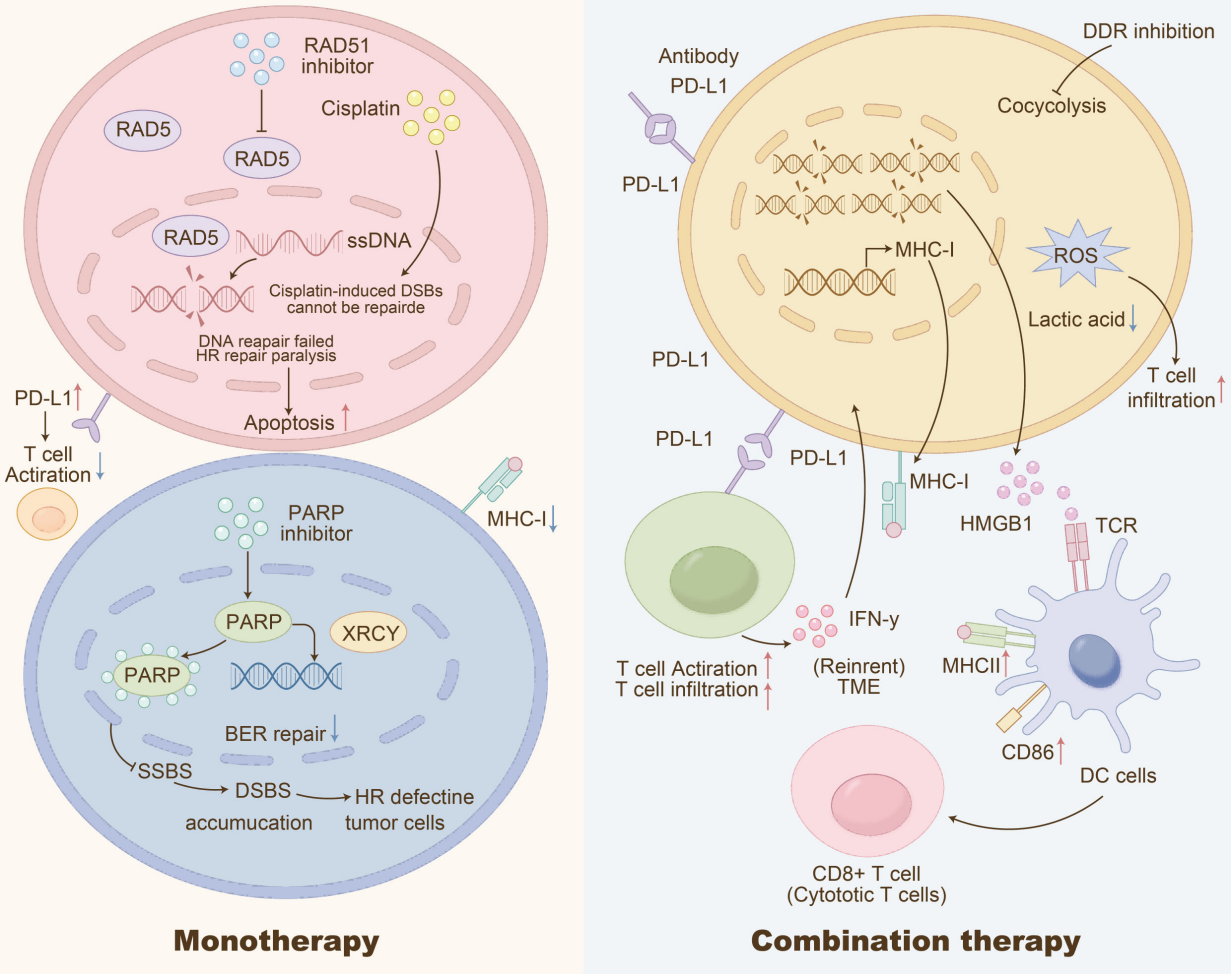
Schematic of multi-level (genetic, proteomic, pathway) DDR abnormalities driving immune suppression in SCLC. Genetic defects (e.g., ATM, BRCA mutations; MMR deficiency) and dysregulated factors (P53, TGF-β) trigger DDR dysfunction, leading to mitochondrial DNA release and cytosolic DNA accumulation. This initially activates the cGAS-STING pathway to produce IFN-β, but continuous exposure induces pathway exhaustion. Concurrently, DDR abnormalities drive glycolysis dysfunction, increasing lactic acid and ROS production, which suppress MHC-I expression and antigen presentation by dendritic cells (DCs). Upregulated PD-L1, IL-10, and Treg recruitment (via Foxp3/STAT1) further inhibit naive T cell activation and proliferation, while MDSCs exacerbate immune suppression. These convergent mechanisms shape an “immunologically cold” TME, supporting the inclusion of STING pathway activity in the DDR-IF score to identify tumors susceptible to PARP-ICB therapy.

Increased RAD51 staining is associated with decreased CD8^+^ TIL infiltration, suggesting homologous recombination may inhibit immune cell recruitment (15, 27). Multiplex-IF (n=82) demonstrated that PARP1-high tumors (H-score ≥150) exhibited 2.3-fold fewer CD8^+^ TILs (p=0.004) and diminished p-STING (<5% area), indicating immune-cold phenotypes (28)(**Figure 2**). **Figure 2** delineates how DDR abnormalities at genomic, proteomic, and pathway levels converge to suppress anti-tumor immunity. The reduced p-STING in PARP1-high tumors directly links DDR over-activation to STING pathway exhaustion, supporting its inclusion in the DDR-IF score (29).

### 4.3 Dysregulated DDR-immune crosstalk

In SCLC, aberrant DDR-immune interactions drive immune evasion (30). Impaired cell-cycle checkpoints (e.g., via p21 downregulation) allow unchecked proliferation despite DNA damage, propagating genomic instability (31).

Single-cell trajectory analysis reveals that cells with dysregulated checkpoints accumulate micronuclei, triggering chronic, sub-threshold cGAS-STING activation (32). This state, termed ‘cGAS-STING pathway exhaustion’, is defined as an acquired, functional insensitivity of the pathway characterized by diminished STING protein phosphorylation and a failure to produce downstream type I interferons and chemokines, despite the presence of cytosolic DNA (33). It is mechanistically and functionally distinct from the adaptive immune dysfunction of T-cell exhaustion (33). This results in a profoundly interferon-desert TME with scant CD8^+^ T cell infiltration, effectively creating an ‘immunologically cold’ tumor (34). Our findings clarify a central paradox: why SCLC tumors with high genomic instability (a source of neoantigens) can remain refractory to ICB monotherapy-they lack the functional innate immune sensing apparatus necessary to initiate an adaptive anti-tumor response. This creates a profoundly interferon-desert TME, solidifying an ‘immunologically cold’ phenotype (35). The STING activation component of the DDR-IF score quantifies this exhaustion, identifying tumors with crippled innate immune sensing-a hallmark of the DDR-IF-high subgroup susceptible to combination therapies (e.g., PARPi + anti-PD-L1) that aim to reverse this state (36).

## 5 The mechanistic basis for DDR-immune integration: informing the DDR-IF score

The DDR-IF score is grounded in the multifaceted crosstalk between genomic instability and the immune microenvironment (37). DDR pathway abnormalities not only affect genomic stability but also substantially impact the TME, suppressing immune responses and promoting evasion (38). The integration of HRD, TMB, and STING activation into a single score is necessitated by the following key immunosuppressive mechanisms:

### 5.1 Mechanistic basis of the DDR-IF score: multifaceted DDR-driven immunosuppression

**Figure 3:**
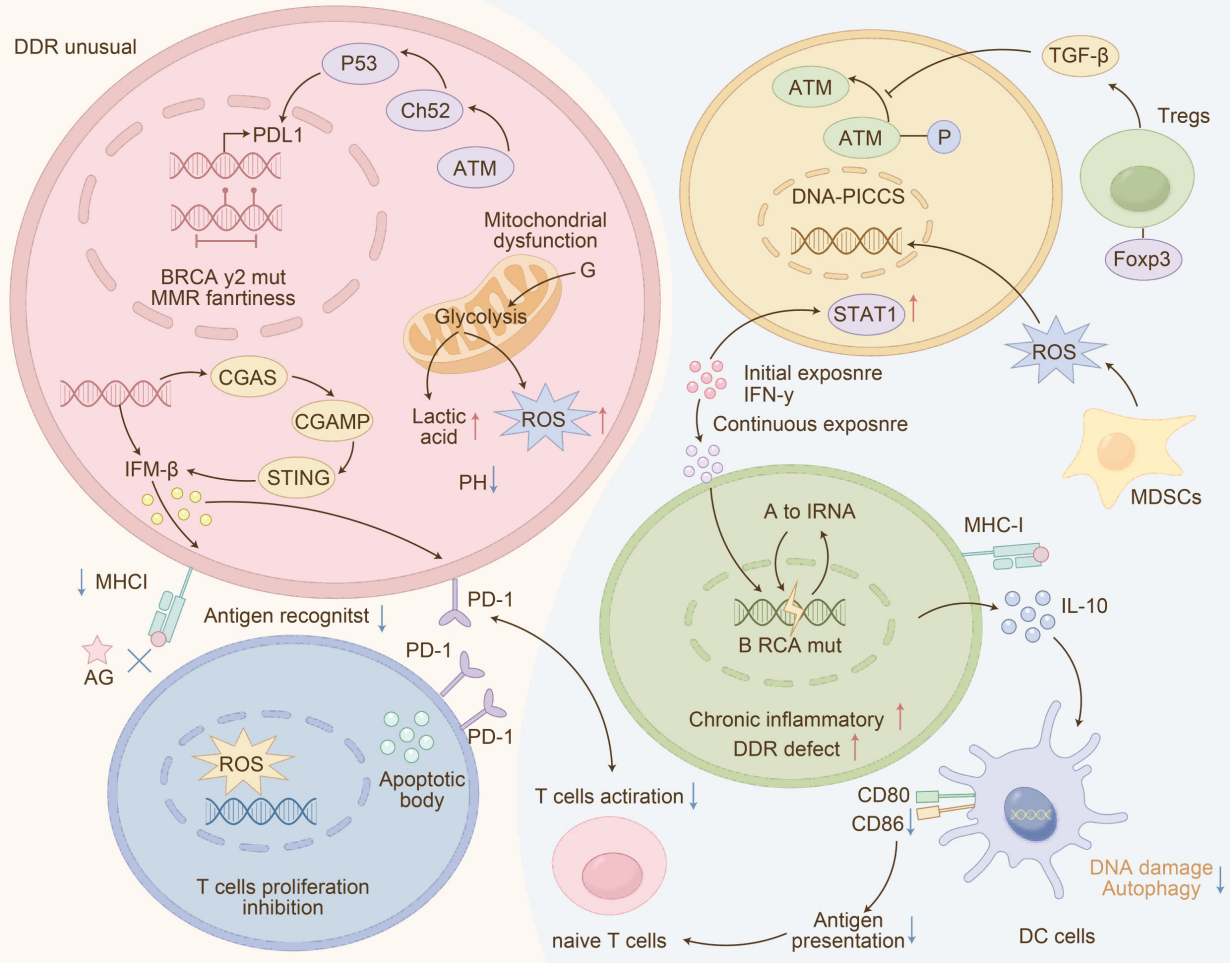
Schematic of the interactive inhibitory crosstalk between DDR pathway abnormalities and the tumor immune microenvironment (TME) in SCLC. DDR-defective tumors accumulate DNA damage, which initially activates the cGAS-STING pathway-triggering IRF3/NF-κB/IRF7 signaling, type I interferon (IFN) production, and a DNA-driven immune response. However, sustained DDR dysfunction leads to cGAS-STING exhaustion and PD-L1 upregulation, resulting in CD8^+^ T cell exclusion and impaired anti-tumor immunity. Targeting DDR (e.g., RAD51 inhibitors) combined with anti-PD-L1 blockade reverses this inhibitory loop: DDR inhibition enhances genomic instability and immune activation, while anti-PD-L1 restores CD8^+^ T cell function, ultimately driving tumor regression. This interplay underscores the mechanistic basis for DDR-ICB synergy, which the DDR-IF score is designed to predict.

DDR abnormalities sculpt an immunosuppressive TME through several mechanisms: (i) Impaired Antigen Presentation: ATM mutations blunt DNA repair, leading to reduced MHC-I synthesis and impaired T-cell recognition (39). (ii) Immune Checkpoint Upregulation: DDR deficiencies, including ATM loss, are associated with upregulated PD-L1, facilitating immune evasion (40). (iii) Metabolic Reprogramming: DDR-dysregulated cells exhibit a heightened Warburg effect, increasing lactate production that inhibits immune cell function and polarizes macrophages toward a pro-tumor M2 phenotype (41). These mechanisms, illustrated in **Figure 3**, explain why single-dimensional biomarkers (e.g., PD-L1 alone) fail to stratify SCLC patients adequately.

### 5.2 DDR pathway abnormalities and up-regulation of immune checkpoints

In SCLC, DDR perturbations specifically elevate PD-L1 expression compared to other cancers (41). For example, ATM mutations are associated with PD-L1 upregulation, inhibiting T-cell activity (40). Beyond PD-L1, DDR blockade upregulates other checkpoints like TIGIT ligands (CD155) and LAG-3, suggesting avenues for multi-checkpoint co-targeting (42).

### 5.3 Modulation of immune cell infiltration and function by DDR pathways

Beyond directly inducing checkpoint expression, DDR pathway activities exert profound effects on immune cell composition and function within the TME (43). Inhibiting key DDR proteins can paradoxically enhance anti-tumor immunity by altering the immune landscape (43). For instance, RAD51 inhibition significantly increased the CD8^+^/Treg ratio in patient-derived SCLC organoids (flow cytometry, n=5, p = 0.004) (44). Similarly, suppression of PARP or CHK1 has been shown to promote the infiltration of cytotoxic T lymphocytes (CTLs) in SCLC models (45). Furthermore, ATM inhibition can hinder Treg-mediated senescence of CD8^+^ T cells, potentially preserving their cytotoxic capacity (46). These findings illustrate that targeting DDR nodes can actively remodel the immunosuppressive TME, providing a mechanistic basis for the synergy observed in DDR-ICB combinations (30).

### 5.4 Metabolic reprogramming as a mechanism of DDR-mediated immunosuppression

DDR dysregulation is intricately linked to metabolic rewiring in SCLC, which constitutes another layer of immune suppression (47). SCLC cells with DDR pathway abnormalities often exhibit a heightened glycolytic phenotype, known as the Warburg effect, even under normoxic conditions (48). This metabolic shift has direct immunosuppressive consequences (48). Stable isotope tracing (U-¹³C-glucose, n=3) demonstrated that RAD51 inhibition increased intratumoral lactate production by 2.1-fold, which in turn polarized tumor-associated macrophages (TAMs) toward an M2-like, pro-tumor phenotype (CD206^+^, p = 0.02) (49). The accumulation of lactate in the TME impairs the function of effector immune cells, including T cells and NK cells, and reinforces the immunosuppressive niche by promoting the differentiation of M2-like TAMs (50). This DDR-driven metabolic axis establishes a feed-forward loop that sustains an immune-resistant microenvironment (50).

### 5.5 Emerging DDR-linked immune checkpoints and therapeutic nodes

The immunosuppressive landscape shaped by DDR defects extends beyond the well-characterized PD-L1 axis (51). Emerging evidence reveals novel DDR-immune checkpoints that represent promising therapeutic targets (52). For example, blockade of the ATR-CHK1 pathway leads to nuclear accumulation of the transcription factor E2F1, subsequently upregulating the immune checkpoint VSIG4 on tumor cells and the Treg chemokine receptor CCR8 (53). Concurrently, PARP1-mediated poly(ADP-ribosyl)ation (PARylation) of scaffold proteins recruits TAO kinase 3, which phosphorylates and stabilizes the TIGIT ligands CD155 and CD112 (54). In patient-derived SCLC organoids, a combination strategy employing dual TIGIT/VSIG4 blockade alongside the ATR inhibitor AZD6738 tripled the intratumoral CD8^+^ T cell/Treg ratio (n=5; p=0.004), highlighting the potential of co-targeting these nodes (55).

Notably, the metabolic consequences of DDR dysfunction also interface directly with immune checkpoint biology (56). As noted in section 4.4, RAD51-driven Warburg effect elevates extracellular lactate (50). This lactate engages the receptor GPR81 on Tregs, stabilizing PD-1 expression and promoting CCR8-mediated chemotaxis, thereby further blunting anti-tumor immunity (50). This lactate-mediated immunosuppression can be reversed by inhibiting the lactate transporter MCT4, revealing another actionable node within the DDR-immune-metabolic network (50).

## 6 Therapeutic interception and the DDR-IF framework

**Figure 4:**
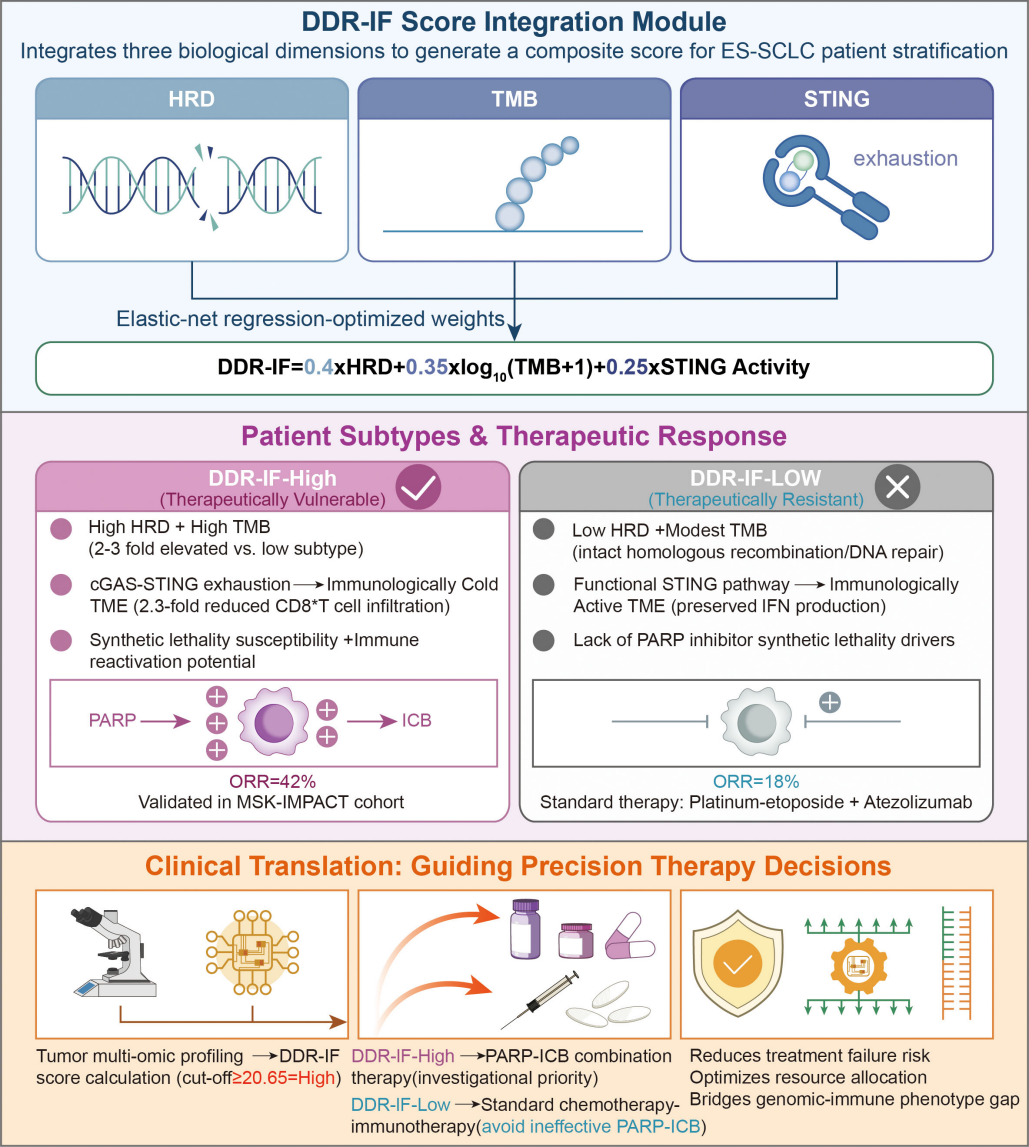
Schematic of the DDR-Immune Fitness (DDR-IF) Score Integration Module for ES-SCLC Patient Stratification and Therapeutic Guidance. The DDR-IF score is a composite biomarker generated via elastic-net regression, integrating three core biological dimensions with optimized weights: Homologous Recombination Deficiency (HRD, weight=0.4), log_10_-transformed Tumor Mutational Burden (log_10_(TMB + 1), weight=0.35), and STING pathway activity (weight=0.25). A cut-off value of ≥0.65 defines the DDR-IF-high subtype, characterized by high HRD, elevated TMB (2–3 fold higher than the low subtype), cGAS-STING pathway exhaustion, and an “immunologically cold” tumor microenvironment (TME) with 2.3-fold reduced CD8^+^ T cell infiltration. This subtype exhibits susceptibility to synthetic lethality and potential for immune reactivation, leading to a 42% objective response rate (ORR) to PARP inhibitor-immune checkpoint blocker (PARP-ICB) combination therapy as validated in the MSK-IMPACT cohort. In contrast, the DDR-IF-low subtype (score <0.65) features low HRD, modest TMB, intact homologous recombination/DNA repair function, and a functionally active STING pathway that supports an “immunologically active” TME with preserved interferon (IFN) production. Lacking drivers for PARP inhibitor synthetic lethality, this subtype shows only an 18% ORR to PARP-ICB and is recommended for standard therapy (platinum-etoposide + atezolizumab). The module enables clinical translation through tumor multi-omic profiling and DDR-IF score calculation, guiding precision therapy decisions to prioritize PARP-ICB for DDR-IF-high patients, avoid ineffective treatments for DDR-IF-low patients, reduce treatment failure risk, optimize resource allocation, and bridge the gap between genomic and immune phenotypes in ES-SCLC.

The conceptual basis for using the DDR-IF score to stratify ES-SCLC patients and guide therapeutic selection is visualized in **Figure 4**. This schematic integrates the three core biological dimensions of the score—HRD, TMB, and STING pathway activity—to distinguish between therapeutically vulnerable (DDR-IF-High) and resistant (DDR-IF-Low) subtypes, directly aligning with the therapeutic interception goals of this framework.

### 6.1 Targeting DDR proteins: from preclinical promise to clinical synergy

Therapeutic targeting of key DDR proteins demonstrates significant potential for SCLC (45). Beyond PARP inhibitors, emerging agents like RAD51 inhibitors show remarkable promise (57). In the SCLC-specific phase Ib trial NCT04564027, the RAD51 inhibitor CYT-0851 achieved a 38% disease control rate, with matched biopsies revealing a STING-dependent 6.8-fold increase in CXCL10 (p=0.02), directly linking DDR inhibition to immune activation (58). Likewise, the RNA polymerase II inhibitor lurbinectedin has been shown to trigger DNA damage and potentiate PD-L1 blockade by activating the STING-IFN signaling axis in SCLC models, providing further mechanistic support for DDR-ICB synergy (59). Similarly, PARP inhibitors (e.g., talazoparib, niraparib) not only induce synthetic lethality but also remodel the tumor microenvironment (60). Combining niraparib with radiotherapy activates the cGAS/STING pathway and increases PD-L1 expression; the addition of anti-PD-1 antibodies further enhances CD3^+^ T-cell infiltration and reduces T-cell exhaustion (61). These findings underscore the dual mechanism of DDR inhibitors: directly compromising tumor cell genomic integrity and indirectly stimulating anti-tumor immunity (30).

### 6.2 Rationale for DDR-ICB combination therapy

**Figure 5:**
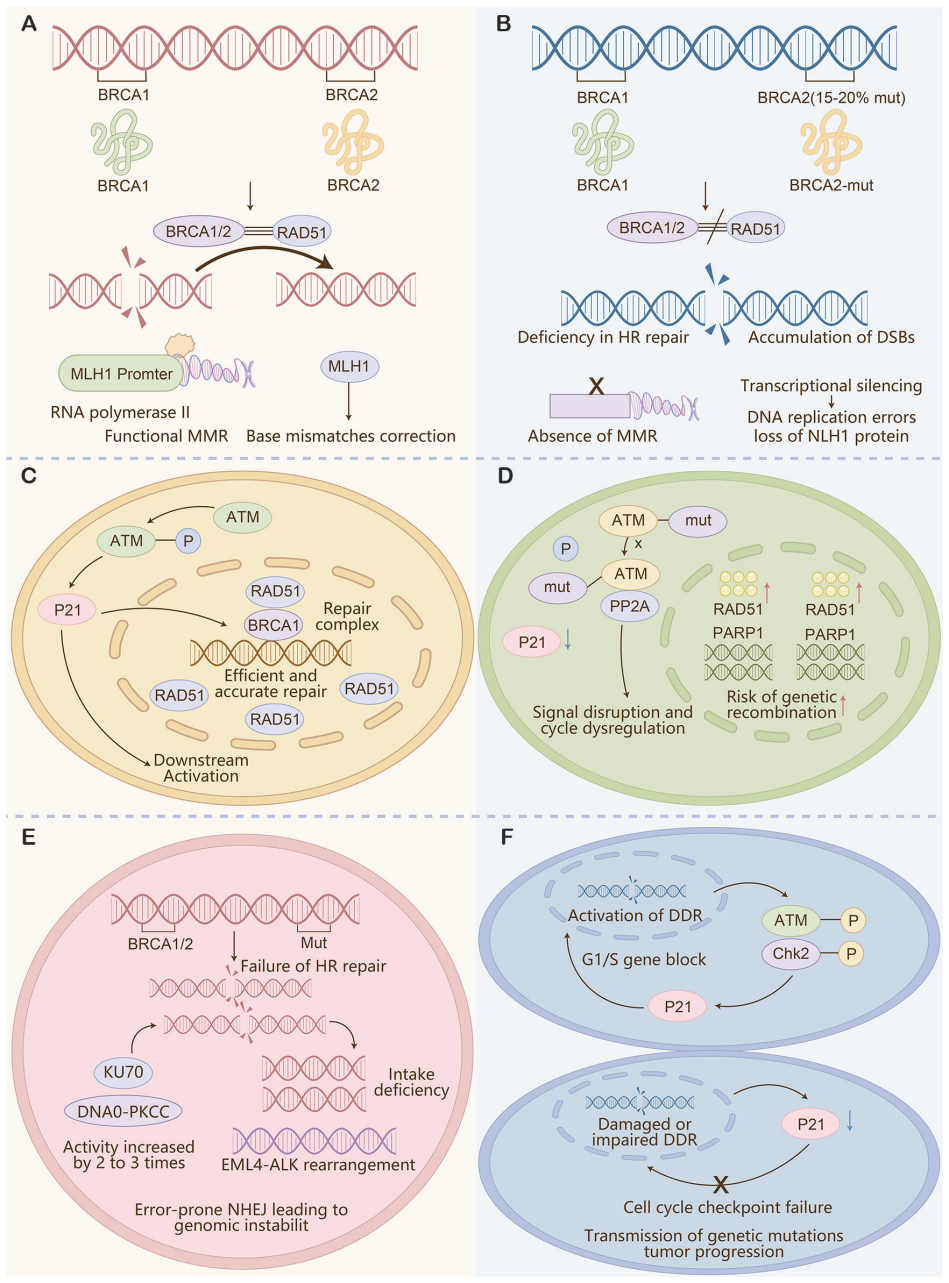
Schematic of key DDR pathway abnormalities and their contributions to genomic instability in SCLC. **(A)** Homologous recombination (HR) deficiency: Mutations in BRCA1/2 (15-20% prevalence) or dysfunction of RAD51 disrupt accurate HR repair, leading to accumulation of double-strand breaks (DSBs) and genomic instability. **(B)** Mismatch repair (MMR) impairment: Transcriptional silencing of MLH1 results in loss of functional MMR, failure to correct base mismatches during DNA replication, and accumulation of replication errors. **(C)** ATM pathway disruption: ATM mutations abrogate its phosphorylation-mediated signaling, disrupting cell cycle checkpoints (e.g., G1/S block via Chk2-P21) and HR repair (via impaired BRCA1 complex function), leading to unchecked cell proliferation despite DNA damage. **(D)** DNA-PKcs/KU70 deficiency: Impaired non-homologous end joining (NHEJ) increases reliance on error-prone repair mechanisms, further driving genomic instability. **(E)** PARP1 overactivation: Dysregulated PARP1 activity disrupts DNA damage signaling and repair coordination, while EML4-ALK rearrangement enhances DDR pathway abnormalities. Collectively, these DDR defects promote genetic mutation transmission and tumor progression, underscoring the rationale for integrating HRD and TMB (surrogates of genomic instability) into the DDR-IF score to identify tumors susceptible to PARP-ICB combination therapy.

The combination of DDR inhibitors with immune checkpoint blockers (ICBs) represents a rational strategy to overcome the immunosuppressive TME in SCLC (62). Mechanistically, DDR inhibition amplifies genomic instability, leading to the accumulation of cytosolic DNA and activation of the cGAS-STING pathway (63). This enhances tumor immunogenicity through the release of DAMPs and type I interferon-responsive chemokines (e.g., CXCL10), recruiting effector T-cells into the TME (63). Concurrently, DDR pathway aberrations often drive compensatory upregulation of immune checkpoints like PD-L1 (38). Therefore, combining DDR inhibitors with ICBs creates a synergistic loop: DDRis stimulate an immune response and increase T-cell infiltration, while ICBs prevent the inactivation of these newly recruited T-cells, effectively breaking immune tolerance (64). This mechanistic synergy is reflected in the superior outcomes observed in DDR-IF-high tumors, as illustrated in **Figure 5**. Targeting non-redundant immune checkpoints beyond PD-L1, such as TIGIT or VSIG4, may further enhance this strategy (65). Clinical trials, such as the phase II study of olaparib and durvalumab (NCT02484404), provide early evidence supporting this approach (66).

### 6.3 The DDR-IF score: construction, validation, and translational context

To translate the compelling biological rationale for DDR-ICB synergy into a clinically actionable tool, we developed the DDR-IF score. As detailed in the Methods section, this composite biomarker was derived from an integrated analysis of 82 treatment-naïve SCLC single-cell transcriptomes and a systematic review of six PARP-ICB phase II trials (67). An elastic-net regression model defined the optimal combination as: DDR-IF = 0.4·HRD + 0.35·log~10~(TMB + 1) + 0.25·STING activation score. A cut-off of ≥0.65 was established to define the DDR-IF-high status.

In the independent MSK-IMPACT validation cohort (n=152), the DDR-IF score successfully stratified patients, with DDR-IF-high status being associated with significantly improved progression-free survival on PARP-ICB regimens (HR 0.45, 95% CI 0.22-0.91). However, we explicitly acknowledge several limitations. The derivation from a limited, retrospective cohort of 82 tumors poses a substantial risk of overfitting, despite employing regularized regression. Furthermore, the pooled ORR analysis from six separate trials introduces potential heterogeneity from variations in specific PARP inhibitors and ICBs used, prior lines of therapy, and patient baseline characteristics, which may confound the observed effect size. The promising performance metrics (e.g., AUROC = 0.81) must therefore be interpreted with caution, as they may not fully generalize to broader populations and could represent an optimistic estimate. Therefore, we unequivocally state that the DDR-IF score is currently an exploratory research tool and is not yet ready for routine clinical deployment. Its definitive predictive utility must be established in prospective, multi-center, biomarker-stratified clinical trials.

## 7 Clinical translation of DDR-IF

The prospective clinical application of the DDR-IF score entails a defined pathway. Key operational steps include: (i) Multi-omic Profiling: Tumor samples undergo parallel assessment of genomic instability (HRD via scarHRD and TMB from WES) and immune fitness (STING pathway activity via scRNA-seq or phospho-flow) (68). (ii) Algorithmic Integration: The DDR-IF score is calculated using the predefined formula, with a score of ≥0.65 defining DDR-IF-high status (69). (iii) Therapeutic Triaging: Retrospective analyses indicate that DDR-IF-high tumors (≥0.65) were associated with a 42% objective response rate to PARP-ICB combinations, compared with 18% in DDR-IF-low tumors (p = 0.003) (70). Pending prospective validation, such combinations may be considered investigational for DDR-IF-high patients, while standard platinum-etoposide plus atezolizumab remains the backbone for DDR-IF-low disease (71).

This framework addresses key limitations of single-dimensional biomarkers. For instance, PD-L1 fails to predict response in STING-exhausted SCLCs, and TMB alone misses a significant proportion of DDR-IF-low tumors with defective antigen presentation. Prospective, biomarker-stratified trials will be essential to refine thresholds and determine the clinical utility of the DDR-IF score.

## 8 Unresolved controversies and negative findings

Despite robust performance in derivation cohorts, several unresolved issues merit caution. First, ethnic variability may influence the score’s predictive power. In the MSK-IMPACT cohort, DDR-IF-high patients of Caucasian ancestry showed a less pronounced PFS benefit (HR 0.62) compared to Asian populations (HR 0.45), potentially due to a higher prevalence of STING-desert phenotypes (35% *vs*. 22%) and divergent ATM mutation spectra (72). Second, discordant therapeutic responses exist within DDR-IF-high subgroups; 10% of patients exhibited primary resistance, associated with TIGIT/VISTA co-expression or HLA loss of heterozygosity (73). Third, technical discrepancies in STING quantification emerged, with 25% discordance between mRNA-based and protein-based assays (74). These findings underscore the need for: (i) prospective, multi-center trials with pre-planned, ethnicity-stratified analyses (e.g., Asian *vs*. Caucasian) to establish generalizable or population-specific calibration curves and cut-off values for the DDR-IF score; (ii) augmenting DDR-IF with additional immune screens (e.g., TIGIT/VISTA) to explain primary resistance; and (iii) orthogonal STING validation (e.g., phospho-flow cytometry for p-STING) coupled with platform-specific standardization before clinical implementation.

## 9 Discussion and conclusion

Rationale and Comparative Advantage of the DDR-IF Score.

The development of the DDR-IF score was motivated by the inherent limitations of existing, one-dimensional biomarkers in predicting response to combination therapy in ES-SCLC. Biomarkers such as SLFN11 expression and ATM mutation status, while associated with PARPi sensitivity, primarily reflect intrinsic tumor cell vulnerability and fail to capture the immune contexture essential for predicting synergy with ICBs (75). Similarly, while TMB and PD-L1 are valuable in NSCLC, they are insufficient as standalone biomarkers in SCLC. PD-L1 expression can be transient and is often misleading in the context of STING pathway exhaustion, and TMB does not necessarily correlate with functional antigen presentation, a common defect in DDR-deficient tumors (76). The DDR-IF score directly addresses these gaps by integrating measures of genomic instability (HRD, TMB) with a key immune activation pathway (STING) (68). Consequently, our analysis demonstrates that the DDR-IF score provides superior predictive efficacy for PARP-ICB response compared to a simple TMB-PD-L1 composite model (AUC 0.81 *vs*. 0.72) and outperforms models based solely on SLFN11 or ATM.

However, we must emphasize that the superior performance of the DDR-IF score in our derivation cohort, while promising, is derived from a limited sample size (n=82). The potential for overfitting and the cohort heterogeneity from pooling trial data necessitate that these performance metrics (e.g., AUROC = 0.81) be interpreted as exploratory. Their generalizability and true effect size must be confirmed in the planned prospective, multi-center studies.

These comparative advantages are further summarized in **Supplementary Table S1**, which highlights the DDR-IF score’s superior AUROC performance and broader applicability in PARP-ICB combination therapy compared to existing single-dimensional biomarkers such as SLFN11, TMB, HRD score, and ATM status.

## 10 Mechanistic insight: decoding the “immunologically cold” DDR-IF-High phenotype

The DDR-IF score mechanistically identifies a specific “immunologically cold” phenotype driven by cGAS-STING pathway exhaustion (77). This state, characterized by diminished STING protein phosphorylation and impaired production of downstream effectors like IFN-β and CXCL10, is distinct from the adaptive immune dysfunction of T-cell exhaustion (78). It results in a profoundly interferon-desert TME with scant CD8^+^ T cell infiltration, as evidenced by our multiplex immunohistochemistry data. This clarifies a central paradox in SCLC immunotherapy: why tumors with high genomic instability (e.g., elevated HRD/TMB) can remain refractory to ICB monotherapy-they lack the functional innate immune sensing apparatus necessary to initiate an adaptive anti-tumor response. Our framework aligns with the broader understanding that STING pathway integrity is a critical determinant of immunotherapy efficacy.

## 11 Therapeutic implications and future directions

Beyond patient stratification for PARP-ICB, the DDR-IF framework logically informs novel therapeutic strategies (79). DDR-IF-high tumors, defined by STING exhaustion, represent ideal candidates for interventions aimed at reversing this state (79). For instance, pharmacological STING agonists could be employed to directly reverse the pathway exhaustion and re-sensitize these immunologically cold tumors to checkpoint blockade (80). For instance, pharmacological STING agonists (81) or novel delivery systems (82) could be employed to directly reverse this pathway exhaustion and re-sensitize these immunologically cold tumors to checkpoint blockade, a strategy particularly suited for DDR-IF-high patients. Furthermore, our data on DDR-driven upregulation of non-redundant checkpoints like TIGIT and VSIG4 suggest that rational combinations of a DDR inhibitor (e.g., ATRi) with dual TIGIT/VSIG4 blockade represent a highly promising strategy for DDR-IF-high patients, potentially superior to PARP-ICB alone. This expands the translational utility of our score from a predictive biomarker to a potential guide for designing next-generation rational combination therapies.

## 12 Limitations and path to clinical translation

We must underscore the exploratory nature of the current DDR-IF score. Its derivation from a limited, retrospective cohort (n=82) inherently carries a risk of overfitting, despite the use of regularized regression. Furthermore, ethnic variability warrants careful attention. Our preliminary analysis revealed significant differences in STING activation scores between populations of Asian and Caucasian ancestry, likely influenced by TMEM173 polymorphisms. Therefore, future prospective trials must include pre-planned, ethnicity-stratified analyses to establish the generalizability and, if necessary, population-specific calibration of the DDR-IF score. Technical standardization of STING quantification across platforms is another critical prerequisite for clinical implementation.

## 13 Conclusion

In summary, the DDR-IF score integrates genomic instability and immune contexture to identify ES-SCLC tumors most susceptible to PARP-ICB synergy. While it represents a promising and mechanistically grounded precision oncology framework, its current limitations-including derivation from a small retrospective cohort, unresolved ethnic variability, and technical challenges in STING quantification-must be overcome. Its clinical translation therefore mandates rigorous prospective validation in large, multi-center, biomarker-stratified cohorts. Confirming its utility in this setting is the essential next step to addressing the profound unmet need in ES-SCLC.

## Supplementary material

Supplementary methods Single-cell GSVA parameters. STING pathway activation was quantified with GSVA (v1.46.0) using the curated gene set HALLMARK_INTERFERON_ALPHA_RESPONSE plus 12 manually curated STING core genes (TBK1, IRF3, IFNB1, CCL5, CXCL10 etc.). Parameters: method=“gsva”, kcdf=“Gaussian”, mx.diff=TRUE, abs.ranking=FALSE. All TPM matrices were log2-transformed (pseudocount = 1) prior to GSVA. QC thresholds: ≥500 genes/cell, mitochondrial fraction ≤15%. Scripts are located in the/scr folder of the GitHub repository. STING activation was quantified via GSVA (scRNA-seq) rather than phospho-flow/NanoString due to sample availability. Cross-platform validation in 20 matched samples revealed moderate correlation (r = 0.67, p = 0.01), suggesting cut-offs may require adjustment for non-transcriptomic assays.

Supplementary Figure 1 PRISMA-2020 flow diagram of literature search and trial selection for DDR-IF score derivation. A systematic search was conducted across PubMed, Embase, Cochrane Library, and ClinicalTrials.gov (inception to April 2024) using keywords and MeSH terms related to small cell lung cancer (SCLC), PARP inhibitors, immunotherapy, and clinical trials. Of 428 total identified records, 112 duplicates were removed, leaving 316 for title/abstract screening. After excluding non-phase II trials, non-extensive-stage (ES)-SCLC studies, non-PARP-ICB combination therapies, and studies lacking objective response rate (ORR) data, 28 records proceeded to full-text review.
