# Supplementary material for: The DDR-immune fitness score: a biomarker for guiding parp and immunotherapy synergy in extensive-stage small cell lung cancer
Source: Front Oncol. 2025 Dec 19;15:1680921. doi: 10.3389/fonc.2025.1680921 (PMC12757875; doi:10.3389/fonc.2025.1680921)
Supplement: Supplementary methods — Single-cell GSVA parameters. STING pathway activation was quantified with GSVA (v1.46.0) using the curated gene set HALLMARK_INTERFERON_ALPHA_RESPONSE plus 12 manually curated STING core genes (TBK1, IRF3, IFNB1, CCL5, CXCL10 etc.). Parameters: method=“gsva”, kcdf=“Gaussian”, mx.diff=TRUE, abs.ranking=FALSE. All TPM matrices were log2-transformed (pseudocount = 1) prior to GSVA. QC thresholds: ≥500 genes/cell, mitochondrial fraction ≤15%. Scripts are located in the/scr folder of the GitHub repository. STING activation was quantified via GSVA (scRNA-seq) rather than phospho-flow/NanoString due to sample availability. Cross-platform validation in 20 matched samples revealed moderate correlation (r = 0.67, p = 0.01), suggesting cut-offs may require adjustment for non-transcriptomic assays. [file Table1.docx]

| **Biomarker** | **Prediction Dimension** | **AUROC (Validation Cohort)** | **Applicable Treatment Scenario** | **Strengths** | **Limitations** |
| --- | --- | --- | --- | --- | --- |
| DDR-IF Score | PARP-ICB Response | 0.75 | PARP Inhibitor + ICB Combination | Integrates DDR and immune features; Optimal predictive performance | Requires multicenter prospective validation |
| SLFN11 | PARP Inhibitor Monotherapy Response | 0.62 | PARP Inhibitor Monotherapy | High specificity for monotherapy | Limited predictive value for combination therapy |
| TMB | ICB Monotherapy Response | 0.58 | ICB Monotherapy | Extensive experience in pan-cancer applications | Weak correlation with response in SCLC |
| HRD Score | PARP Inhibitor Monotherapy Response | 0.60 | PARP Inhibitor Monotherapy | Reflects genomic instability | Does not integrate immune features; Insufficient for combination therapy prediction |
| ATM Status | PARP Inhibitor Response | - (Low Incidence) | PARP Inhibitor Monotherapy/Combination | Mechanistically clear (homologous recombination deficiency) | Incidence only 5%-8% in SCLC; Insufficient sample size |

**Table S1. Performance Comparison of DDR-IF and Existing Biomarkers**
